# TRAIL suppresses gut inflammation and inhibits colitogeic T-cell activation in experimental colitis via an apoptosis-independent pathway

**DOI:** 10.1038/s41385-019-0168-y

**Published:** 2019-05-11

**Authors:** I. T. Chyuan, H. F. Tsai, C. S. Wu, P. N. Hsu

**Affiliations:** 1 Department of Internal Medicine, Cathay General Hospital, Taipei, Taiwan; 2 Department of Medical Research, Cathay General Hospital, Taipei, Taiwan; 3 School of Medicine, College of Medicine, Fu Jen Catholic University, New Taipei City, Taiwan; 4 Graduate Institute of Clinical Medicine, College of Medicine, Taipei Medical University, Taipei, Taiwan; 5 Department of Internal Medicine, Taipei Medical University Shuang Ho Hospital, New Taipei City, Taiwan; 6 Division of Rheumatology, Department of Internal Medicine, Far Eastern Memorial Hospital, New Taipei City, Taiwan; 7 Graduate Institute of Immunology, College of Medicine, National Taiwan University, Taipei, Taiwan; 8 Department of Internal Medicine, National Taiwan University Hospital, Taipei, Taiwan

## Abstract

Tumor necrosis factor-related apoptosis-inducing ligand (TRAIL) induces cell apoptosis by transducing apoptosis signals. Recently, accumulating evidence demonstrated that TRAIL regulates autoimmune inflammation and immune cell homeostasis in several autoimmune animal models, suggesting a novel immunoregulatory role of TRAIL in autoimmune diseases. However, the impact of TRAIL in inflammatory bowel disease is yet undefined. This study is to address the therapeutic effects and immunoregulatory role of TRAIL in autoimmune gut inflammation. We demonstrated herein that TRAIL significantly suppressed gut inflammation and reduced the severity of colitis in a dextran sodium sulfate (DSS)-induced colitis model. Suppression of gut inflammation was not due to induction of apoptosis in colonic T cells, dendritic cells, or epithelium cells by TRAIL. In contrast, TRAIL directly inhibited activation of colitogenic T cells and development of gut inflammation in an adoptive transfer-induced colitis model. The anti-inflammatory effects of TRAIL on colitis were abolished when T cells from TRAIL receptor (TRAIL-R) knockout mice were adoptively transferred, suggesting that TRAIL regulates autoreactive colitogenic T-cell activation in the development of gut inflammation. Our results demonstrate that TRAIL effectively inhibited colonic T-cell activation and suppressed autoimmune colitis, suggesting a potential therapeutic application of TRAIL in human inflammatory bowel disease.

## Introduction

The tumor necrosis factor (TNF)-related apoptosis-inducing ligand (TRAIL) is a member of the TNF superfamily that preferentially induces cell apoptosis in a variety of transformed cell lines.^1^ TRAIL induces cell apoptosis via binding to its cognate death receptor, DR4 and DR5, and transduces apoptosis signaling, resulting in activation of a caspase cascade.^2,3^ In mice, there is only one death-inducing TRAIL receptor (TRAIL-R), which is homologous to human DR5.^4^ TRAIL and TRAIL receptors are constitutively expressed in various tissues ^1,2,4,5^; however, most normal primary cells are resistant to TRAIL-induced cell death.^6^ Although cancer killing was initially suggested, the actual biological role of TRAIL is still not clear. Recent accumulating evidence in several autoimmune animal models suggests an immunoregulatory role of TRAIL in controlling inflammation in autoimmune diseases. In autoimmune arthritis, TRAIL blockade^7^ or a TRAIL deficiency ^8^ enhanced disease activity and increased joint inflammation. Similar findings were also observed in mice with experimental autoimmune encephalomyelitis (EAE).^9^ Recent studies further demonstrated that administration of TRAIL is effective in suppressing autoimmune inflammation via an apoptosis-independent pathway.^10,11^ All these results imply a novel immunoregulatory role of TRAIL in autoimmune diseases.

Inflammatory bowel disease (IBD) is an autoimmune disease that results from excessive immune responses to the intestinal microbiota, triggered by a damaged epithelial barrier, changes in the composition of the intestinal microflora, and increased effector T-cell function.^12–15^ A previous study demonstrated that TRAIL-induced human intestinal epithelial cell apoptosis under inflammatory conditions, while it did not induce enterocyte apoptosis in normal noninflammatory conditions,^16^ suggesting that TRAIL contributes to the pathogenesis of gut inflammation. In addition, TRAIL-R knockout (KO) mice exhibited a higher incidence of dextran sodium sulfate (DSS)-induced colitis,^17^ implying that the TRAIL/TRAIL-R pathway may be involved in the regulation of autoimmune colitis. However, the role of TRAIL in IBD is yet undefined.

In this study, we investigated the immunoregulatory role and potential therapeutic application of TRAIL in autoimmune colitis models. We demonstrate herein that TRAIL effectively suppressed gut inflammation via an apoptosis-independent pathway and directly inhibited activation of colitogenic T cells via interaction with TRAIL-R. Our study indicates a novel immunoregulatory role of TRAIL in colonic inflammation and mucosal immunity. It also implies a potential therapeutic approach to IBD.

## Results

### TRAIL inhibits disease severity and gut inflammation in DSS-induced colitis

**Fig. 1:**
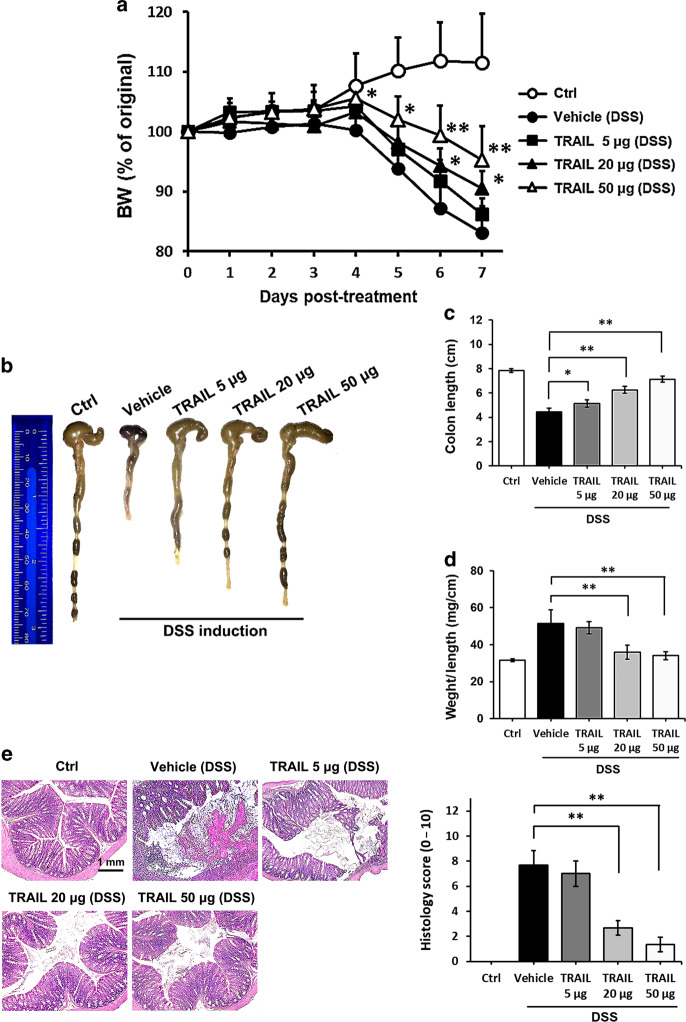
TRAIL inhibited disease severity and gut inflammation in DSS-induced colitis. In the group with DSS-induced colitis, C57BL/6 mice were fed 2.5% DSS in sterilized drinking water for 5 days (days 0–5), followed by 2 days of normal water (days 6 and 7) and treated with either the vehicle (200 μl/mouse/day, i.p.) or different dosages of TRAIL (5, 20, and 50 μg/mouse/day, i.p.) from day 0. Control mice were given normal drinking water only. **a** Body weight changes (% original body weight) in each group were recorded, and results are presented as the mean ± SD (*n* = 15 in each group). * *p* < 0.05, ** *p* < 0.01 by Student’s *t* test, compared to DSS-induced colitis mice treated with vehicle. **b** Representative colon lengths in each group are shown at the end of treatment (day 7). **c** Quantitative colon length and **d** the ratio of the colon weight to the length in each group are shown at the end of treatment (day 7). Data are presented as the mean ± SD (*n* = 15 for each group). * *p* < 0.05, ** *p* < 0.01 by Student’s *t* test. **e** Representative H&E staining of colon tissues (distal part of the colon, day 7) from each group is shown (left panel) and was quantified by a histological injury score (right panel). **p < 0.01 by Student’s *t* test. Data are representative of at least three independent experiments

In order to explore possible roles of TRAIL in the development and pathogenesis of autoimmune colitis and the therapeutic potential of TRAIL in IBD, TRAIL was administered to mice with DSS-induced colitis. Soluble recombinant TRAIL was intraperitoneally injected at 5, 20, and 50 μg/mouse every day in mice treated with DSS. As illustrated in Fig. 1, marked body weight loss developed in vehicle-treated mice on day 5 post-DSS induction. In contrast, TRAIL treatment of mice with DSS induction potently protected them against body weight loss in a dose-dependent manner (Fig. 1a). Furthermore, on day 7 post-DSS induction, the colon lengths of TRAIL-treated mice were also significantly longer than those of vehicle-treated mice, and the effect was dose-dependent (Fig. 1b–d). Histologically, vehicle-treated mice showed severe epithelial damage, goblet cell loss, and dense cellular infiltrates; however, little tissue damage and inflammation was seen in TRAIL-treated mice (Fig. 1e). Taken together, our results indicate that TRAIL significantly reduced the development and severity of gut inflammation in mice with DSS-induced colitis.

### TRAIL significantly inhibits the production of proinflammatory cytokines and chemokines in DSS-induced colitis

**Fig. 2:**
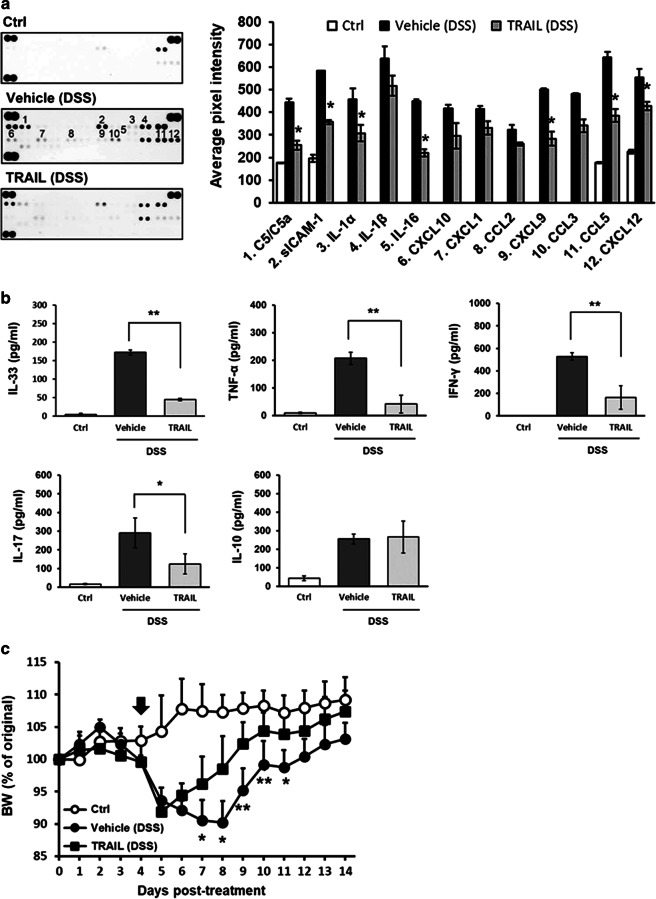
TRAIL inhibited a panel of proinflammatory cytokines and chemokines in inflamed colon tissues of mice with DSS-induced colitis. **a** In the group with DSS-induced colitis, C57BL/6 mice were fed 2.5% DSS in sterilized drinking water for 5 days (days 0–5), followed by 2 days of normal water (days 6 and 7) and treated with either vehicle (200 μl/mouse/day, i.p.) or TRAIL (50 μg/mouse/day, i.p.) from day 0. Control mice were given normal drinking water only (*n* = 15 for each group). On day 7, colon tissues were moved from each group, and tissue lysates were analyzed by a cytokine array. Representative cytokine array data are shown (left panel) and quantified (right panel). Data are presented as the mean ± SD of triplicate samples. *p < 0.05 by Student’s *t* test, compared to DSS-induced colitis mice treated with the vehicle. **b** On day 7, colon tissue lysates were prepared from each group, and specific cytokine levels, including interleukin (IL)-33, TNF-α, interferon (IFN)-γ, IL-17, and IL-10 were assayed by ELISAs. Data are presented as the mean ± SD of triplicate samples. *p < 0.05, **p < 0.01 by Student’s *t* test. Data are representative of at least three independent experiments. **c** In the group with DSS-induced colitis, C57BL/6 mice were fed 2.5% DSS in sterilized drinking water for 5 days (days 0–5), followed by 2 days of normal water (days 6 and 7) and treated with either vehicle (200 μl/mouse/day, i.p.) or TRAIL (50 μg/mouse/day, i.p.) from day 4. Control mice were given normal drinking water only. Body weight changes (% original body weight) in each group were recorded, and results are presented as the mean ± SD (*n* = 15 in each group). *p < 0.05, **p < 0.01 by Student’s *t* test, compared to DSS-induced colitis mice treated with TRAIL

To further investigate whether TRAIL inhibits the inflammatory process by regulating the production of proinflammatory cytokines and chemokines, we measured a panel of cytokines and chemokines from inflamed colon tissues on day 7 in mice with DSS-induced colitis under vehicle or TRAIL treatment. As illustrated in Fig. 2, levels of proinflammatory chemokines, including CXCL9 (MIG), CCL5 (RANTES), and CXCL12 (SDF-1), as well as other chemoattractants, including C5a and IL-16, were much lower in TRAIL-treated mice with DSS induction, compared to those in vehicle-treated control mice (Fig. 2a). In addition, similar results were observed for proinflammatory cytokines, including TNF-α, IFN-γ, IL-17, IL-1α, and IL-33 (Fig. 2b). Furthermore, there were reduced gut infiltrating macrophages and T cells in TRAIL-treated colitis mice; and the macrophage-derived cytokine, TNF-α, and T cell-derived cytokine, IL-17, were also significantly decreased (Supplementary Fig. S1). All these results indicate that TRAIL inhibits both the recruitment of innate and adaptive immune cells into the inflamed guts. Taken together, these results demonstrated that TRAIL significantly reduced colonic proinflammatory cytokine and chemokine production in DSS-induced colitis. To further address the therapeutic application of TRAIL in IBDs, we administered TRAIL in mice already showing initial clinical signs of colitis after DSS induction. As shown in Fig. 2c, TRAIL was administrated from day 4 when the first clinical signs of colitis developed in mice with DSS induction. The results demonstrated treatment with TRAIL significantly recovered the colitis and reduced the disease severity, indicating TRAIL has a therapeutic potential in IBDs.

### Suppression of gut inflammation by TRAIL is dependent on interactions with TRAIL-R

**Fig. 3:**
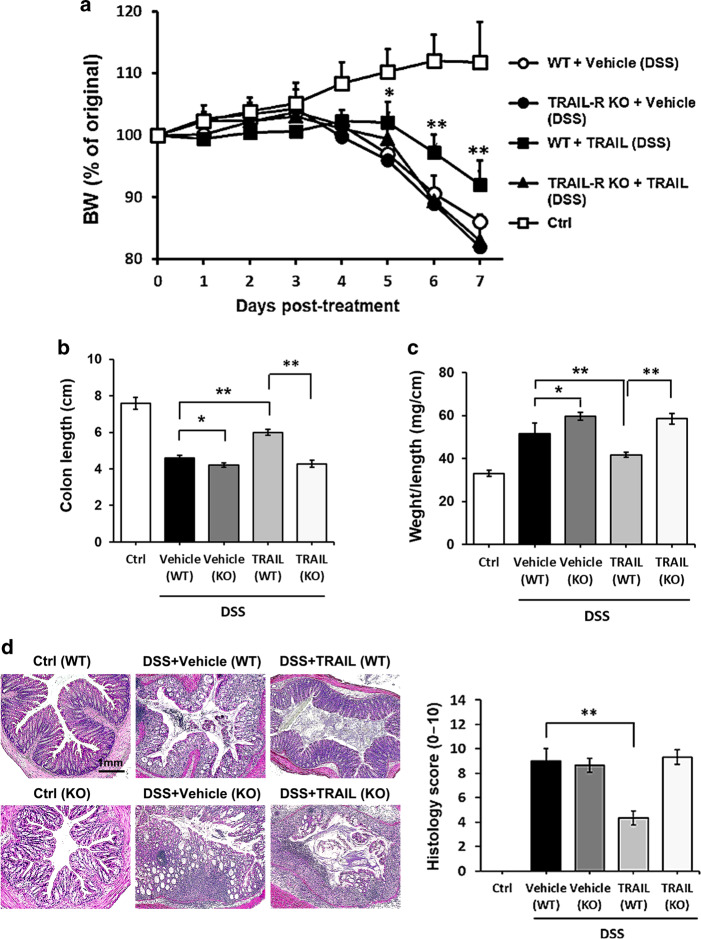
Inhibition of gut inflammation by TRAIL is through TRAIL receptor (TRAIL-R). Wild-type C57BL/6 (WT) mice and TRAIL-R knockout (TRAIL-R KO) mice were fed 2.5% DSS in sterilized drinking water for 5 days (days 0–5), followed by 2 days of normal water (days 6 and 7) and treated with either the vehicle (200 μl/mouse/day, i.p.) or TRAIL (50 μg/mouse/day, i.p.) from day 0. Control (WT) mice were given normal drinking water only. **a** Body weight changes (% original body weight) in each group were recorded and are presented as the mean ± SD (*n* = 15 in each group). *p < 0.05 by Mann–Whitney *U* test, compared to WT mice treated with the vehicle. **b** Quantitative colon length and **c** the ratio of the colon weight to length of each group are shown at the end of treatment (day 7). Data are presented as the mean ± SD. *p < 0.05, **p < 0.01 by Student’s *t* test. **d** Representative H&E staining of colon tissues (distal part of colon, day 7) from each group is shown (left panel) and quantified by a histological injury score (right panel). **p < 0.01 by Student’s *t* test

To confirm that the anti-inflammatory effect on DSS-induced colitis by TRAIL is dependent on TRAIL/TRAIL-R interactions, we further used TRAIL-R KO mice to evaluate the anti-inflammatory effects of TRAIL on DSS-induced colitis. Results in Fig. 3 demonstrate that body weight loss (Fig. 3a) and gut inflammation (Fig. 3b–d) in TRAIL-R KO mice were more severe than those of the WT controls with DSS-induced colitis. Moreover, the anti-inflammatory effects of TRAIL, and the TRAIL-induced inhibition of recruitment of innate and adaptive immune cells into the inflamed guts on mice with DSS induction were abolished in TRAIL-R KO mice (Fig. 3 and Supplementary Fig. S1). These results indicated that TRAIL-mediated inhibition of gut inflammation is dependent on interactions with TRAIL-R.

### Suppression of gut inflammation by TRAIL is not due to induction of apoptosis in inflammatory cells

**Fig. 4:**
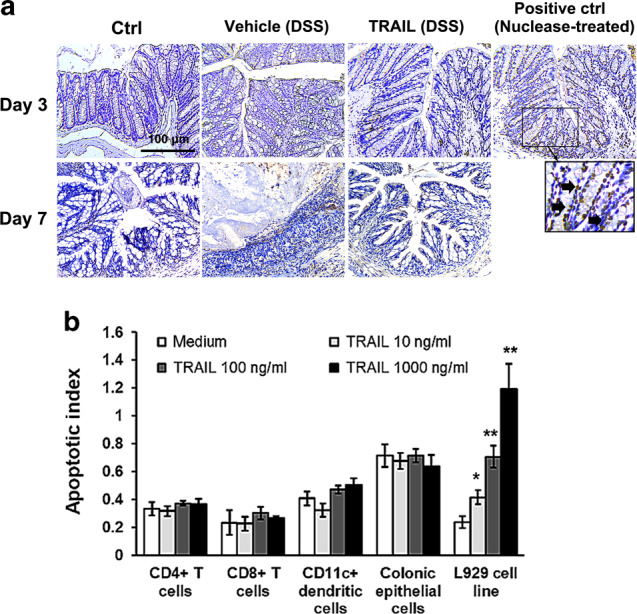
TRAIL does not enhance cell apoptosis in the inflamed colon of mice with DSS-induced colitis. **a** In the group with DSS-induced colitis, C57BL/6 mice were fed 2.5% DSS in sterilized drinking water for 5 days (days 0–5), followed by 2 days of normal water (days 6 and 7) and treated with either vehicle (200 μl/mouse/day, i.p.) or the TRAIL (50 μg/mouse/day, i.p.) from day 0. Control mice were given normal drinking water only. Terminal deoxynucleotidyl transferase dUTP nick-end labeling (TUNEL) staining of the distal colon tissue from each group was carried out on day 3 and day 7. Positive controls were colon tissue slides from control mice pretreated with DNase I before TUNEL staining. TUNEL^+^ cells (arrow) indicate apoptotic cells. **b** C57BL/6 mice were fed 2.5% DSS in sterilized drinking water for 5 days (days 0–5), followed by 2 days of normal water (days 6 and 7). On day 7, colon tissues were collected and primary CD4^+^ T cells, CD8^+^ T cells, CD11c^+^ dendritic cells, and colonic epithelial cells from colon tissues were isolated, cultured in a 96-well plate (10^4^ cells per well), and treated with various concentrations of TRAIL for 24 h. The L292 cells were cultured and treated with TRAIL in the same manner. Cell lysates were collected and quantified using apoptotic ELISAs. Data are presented as the mean ± SD of triplicate samples. *p < 0.05, **p < 0.01 by Student’s *t* test, compared to the L929 cells without TRAIL treatment. Data are representative of three independent experiments

TRAIL induces cell apoptosis by binding to the cognate death receptor via transducing a caspase-mediated signaling cascade. To determine whether the anti-inflammatory effects on colitis by TRAIL occur through promoting cell apoptosis, we detected apoptotic cells within colon tissues in mice with DSS-induced colitis in the presence and absence of TRAIL. As shown in Fig. 4, there were only minimal apoptotic cells detected within inflamed colon tissues, and no enhancement of apoptotic cells within colon tissues was observed compared to the vehicle- and TRAIL-treated groups on day 3 (earlier stage) or on day 7 (later stage) post-DSS induction (Fig. 4a). We further isolated primary immune cells, including CD4^+^ T cells, CD8^+^ T cells, CD11c^+^ dendritic cells, and colonic epithelial cells, from inflamed colon tissues of mice with DSS induction on day 7 to determine whether they were susceptible to TRAIL-induced apoptosis ex vivo (Fig. 4b). Results demonstrated that no significant cell apoptosis was induced among these primary cells after being treated with different doses of TRAIL. In contrast, TRAIL induced a significant amount of cell apoptosis in the L929 mouse fibroblast cell line in a dose-dependent manner. These results indicate that the anti-inflammatory effect on DSS-induced colitis by TRAIL is not due to inducing cell apoptosis within inflamed colon tissues.

### TRAIL suppresses colitogenic T-cell activation to prevent the development of autoimmune colitis

**Fig. 5:**
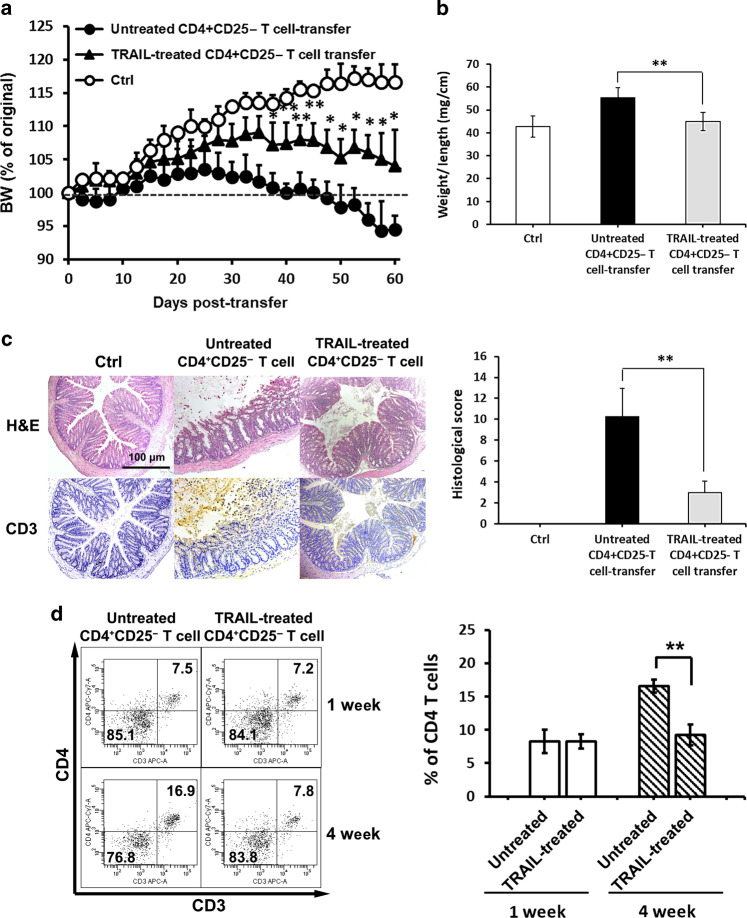
Treatment of colitogenic T cells with TRAIL reduces disease activity and gut inflammation in adoptive transfer-induced colitis. Isolated splenic CD4^+^CD25^−^ T cells from C57BL/6 mice were stimulated with anti-CD3 (1 µg/ml) and anti-CD28 (1 µg/ml) antibodies in the presence or absence of TRAIL (10 µg/ml) for 24 h and adoptively transferred into Rag1 knockout (KO) mice (5 × 10^5^ CD4^+^ T cells/recipient). **a** Adoptive transfer recipients were monitored daily for body weight changes. The mean percentage body weight change ± SD is presented (*n* = 10 for each group). *p < 0.05, **p < 0.01 by Mann–Whitney *U* test. **b** Ratio of the colon weight to length from each group is shown at the end of treatment (day 60). Data are presented as the mean ± SD (*n* = 15 for each group). *p < 0.05, **p < 0.01 by Student’s *t* test. **c** Colon tissues were isolated and examined histologically from each group on day 60 after adoptive transfer. Cross-sections of paraffin-embedded distal colon tissues were stained with hematoxylin and eosin (**h**, **e**) and an anti-CD3 immunohistochemical antibody (left panel). Staining is representative of sections taken from ten mice per group. Arrows indicate inflammatory cells. Arrowheads indicate CD3^+^ T cells. H&E staining of colon tissues were quantified by a histological injury score (right panel). **p < 0.01 by Student’s *t* test. **d** On 1-week and 4-week after adoptive transfer, colonic laminar propria (LP) cells were isolated from the mice of the indicated groups, stained with anti-CD3 and anti-CD4 Ab, and analyzed by flow cytometry. Data are representative of three independent experiments (left panel) and the percentages of CD4^+^ T cells were quantified (right panel). **p < 0.01 by Student’s *t* test

Accumulating evidence implies that TRAIL inhibits T cell activation via an apoptosis-independent pathway.^10,18^ Our recent report demonstrated that TRAIL could inhibit activation of autoantigen-reactive T cells during the development of autoimmune EAE.^11^ In addition, TRAIL directly inhibited T-cell activation and suppressed T-cell receptor signaling (Supplementary Figs. S2 and S3). Moreover, suppression of T-cell activation by TRAIL in the inflamed guts were completely abolished in T cells from TRAIL-R KO (Supplementary Fig. S4). All these results suggest TRAIL may inhibit autoimmune colitis via inhibiting T-cell activation. To further investigate whether TRAIL directly inhibits colitogenic T cells in the development of autoimmune colitis, we used an adoptive transfer colitis model to determine the ability of TRAIL-treated T cells to affect the development of colitis. Splenic CD4^+^CD25^−^ T cells were isolated and treated with TRAIL ex vivo and then were adoptively transferred into Rag1 KO recipient mice. As results shown in Fig. 5, after transfer of vehicle-treated colitogenic T cells, the recipient mice developed prompt body weight loss after day 28 postadoptive transfer, and a persistent decrease in body weight was seen over the experimental course (Fig. 5a). In contrast, when TRAIL-treated colitogenic T cells were adoptively transferred, the recipient mice showed minimal body weight loss over the entire experimental course. On day 60 postadoptive transfer, the colons of recipient mice with TRAIL-treated colitogenic T cells were also significantly longer than those in the vehicle-treated group (Fig. 5b). Furthermore, in the histopathological analysis, recipient mice into which TRAIL-treated colitogenic T cells had been adoptively transferred showed significantly suppressed gut inflammation and reduced T-cell infiltration in colon tissues compared to the control group (Fig. 5c). There are no differences in the homing/survival and repopulation of TRAIL-treated and untreated CD4 T cells after transfer in RAG mice (Fig. 5d). Therefore, reduced engraftment is not the reason for decreased colitis in adoptive transferal of TRAIL-treated CD4 T cells. Taken together, our results demonstrated that TRAIL suppressed colitogenic T-cell activation, and prevented development of gut inflammation in this transfer-induced colitis model. These results indicate that TRAIL directly inhibits colitogenic T cells in the development of colon inflammation.

### TRAIL-mediated suppression of colitogenic T-cell activation and inhibition of colitis are dependent on TRAIL-R

**Fig. 6:**
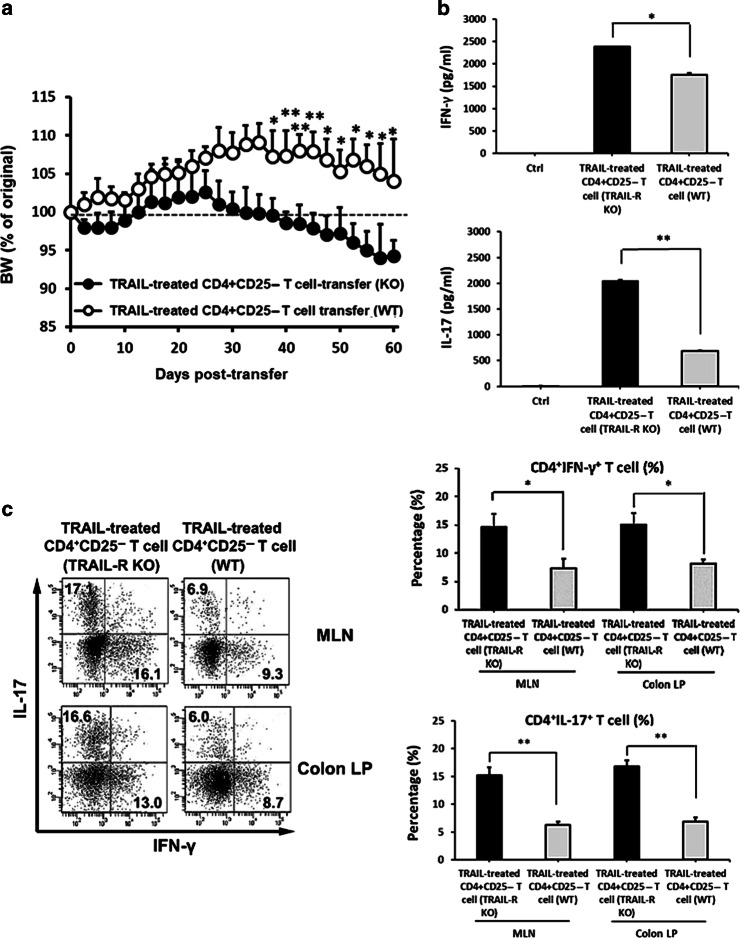
TRAIL-mediated suppression of colitogenic T-cell activation and inhibition of colitis are dependent on TRAIL-R. Isolated splenic CD4^+^CD25^−^ T cells from wild-type (WT) or TRAIL-R knockout (KO) mice were stimulated with anti-CD3 (1 µg/ml) and anti-CD28 (1 µg/ml) antibodies in the presence of TRAIL (10 µg/ml) for 24 h and adoptively transferred into Rag1 KO mice (5 × 10^5^ CD4^+^ T cells/recipient). **a** Adoptive transfer recipients were monitored daily for body weight changes. The mean percentage of body weight change ± SD is presented (*n* = 10 for each group). *p < 0.05, **p < 0.01 by Mann–Whitney *U* test. **b** On day 60 after adoptive transfer, colon tissues from each group were collected, and cytokine levels from tissue lysates were assayed by an ELISA. *p < 0.05, **p < 0.01 by Student’s *t* test. **c** CD4^+^ T cells from mesenteric lymph nodes (MLNs) and colon lamina propria (LP) of each group were isolated on day 60 after adoptive transfer and restimulated with PMA/ionomycin in the presence of GolgiSTOP for 5 h. Interleukin (IL)-17 and interferon (IFN)-γ production by MLN and colon LP CD4^+^ cells were measured by intracellular cytokine staining. Data are representative of three independent experiments (left panel). Percentages of CD4^+^ IL-17^+^ and CD4^+^ IFN-γ^+^ cells were quantified (right panel). *p < 0.05, **p < 0.01 by Student’s *t* test

To further confirm that TRAIL-mediated suppression of colitogenic T cells in the development of colitis is via the TRAIL-R, we adoptively transferred TRAIL-treated splenic CD4^+^CD25^−^ T cells from WT and TRAIL-R KO mice into Rag1 KO mice to induce colitis. As shown in Fig. 6a, TRAIL-mediated suppression of colitogenic T cells from WT mice in the development of colitis was abolished when T cells were adoptively transferred from TRAIL-R KO mice. In addition, TRAIL-mediated suppression of colonic cytokine production was also reversed in TRAIL-R KO mice on day 60 postadoptive transfer (Fig. 6b). Furthermore, mice which received TRAIL-treated colitogenic T cells developed much milder gut inflammation with very few IL-17- and IFN-γ-producing T cells infiltrated into gut tissues compared to the control group; while this protective effect was abolished when mice received TRAIL-treated T cells adoptively transferred from TRAIL-R KO mice (Fig. 6c). These results indicate that TRAIL-mediated suppression of colitogenic T cell activation and inhibition of colitis are TRAIL-R dependent.

## Discussion

In this study, we demonstrated that TRAIL directly inhibited gut inflammation and reduced the severity of colitis in animal models of autoimmune colitis, and these effects were dependent on the TRAIL-R. Furthermore, the suppression of gut inflammation was not due to induction of apoptosis in colonic T cells and other immune cells or epithelium cells by TRAIL. In contrast, TRAIL directly inhibited activation of colitogenic T cells and development of gut inflammation in an adoptive transfer-induced colitis model, indicating a novel role for TRAIL in regulating autoreactive T-cell activation and modulating autoimmune colitis.

TRAIL has long been regarded as an apoptosis-inducing ligand and has been extensively investigated in tumor killing. Accumulating evidence has demonstrated that in addition to triggering apoptosis, TRAIL can modulate immune responses in autoimmune diseases.^7,9,19^ Although the mechanisms by which TRAIL inhibits autoimmune inflammation remain to be elucidated, previous studies explained how the TRAIL-mediated anti-inflammatory effect was presumably due to its proapoptotic activity by triggering apoptosis of inflammatory cells.^20–22^ However, recent reports and our previous studies demonstrated that TRAIL does not promote inflammatory cell apoptosis in several autoimmune animal models, and the anti-inflammatory effect of TRAIL is via an apoptosis-independent pathway.^7,10,11,23^ In agreement with these findings, in this study, we further demonstrated that TRAIL directly inhibited gut inflammation and reduced the severity of colitis in animal models of autoimmune colitis, and the TRAIL-mediated suppression of gut inflammation was not due to induction of apoptosis in inflammatory cells, indicating a novel immunoregulatory role for TRAIL in modulating autoimmune inflammation. These results also imply the potential therapeutic potential of TRAIL in treating human autoimmune diseases.

IBD is a chronic autoimmune inflammatory condition characterized by severe inflammation and mucosal destruction in the intestines. Although the etiology remains unclear, it is now believed that colitis results from excessive immune responses to the intestinal microbiota which are triggered by increased activity of effector T cells along with changes in the composition of the intestinal microflora, and epithelial barrier damage.^12–15,24,25^ Both TRAIL and TRAIL-R were shown to be expressed on intestinal tissues,^26^ but it is not known whether TRAIL plays a role in colonic inflammation. A previous in vitro study demonstrated that TRAIL-induced human intestinal epithelial cell apoptosis under inflammatory conditions, while not inducing enterocyte apoptosis in normal noninflammatory conditions,^16^ suggesting that TRAIL contributes to the pathogenesis of gut inflammation. A recent study by Faletti et al.^27^, implies that TRAIL alone is not sufficient to induce apoptosis in type1 cells; however, it could synergize with other apoptosis triggers to efficiently induce apoptosis in responsive cells. In the present study, we clearly demonstrated that TRAIL directly inhibited gut inflammation and disease severity in DSS-induced colitis. Moreover, the inhibition of gut inflammation by TRAIL was not due to the induction of apoptosis in T cells, dendritic cells, or colonic epithelial cells. The inhibition of autoimmune inflammation via TRAIL/TRAIL-R pathway was observed in several genetically deficient animal models.^8,10,11,28^ In an autoimmune colitis model, TRAIL-R KO mice were also more susceptible to DSS-induced colitis, and we further demonstrated that TRAIL-mediated suppression of gut inflammation was dependent on TRAIL-R. These results indicated that TRAIL-mediated inhibition of gut inflammation is dependent on interactions with the TRAIL-R.

Our results demonstrated that TRAIL suppressed gut inflammation and reduced disease severity in DSS-induced colitis. Moreover, adoptive transfer of TRAIL-treated T cells reduced development of bowel inflammation and colitis in a T-cell transfer colitis model, indicating that TRAIL suppressed colitogenic T-cell activation and prevented the subsequent development of autoimmune colitis. In addition, adoptive transferal of TRAIL-treated T cells developed a later onset and less severe colitis (Supplementary Fig. S5), suggesting TRAIL suppresses colitogenic T-cell activation and further inhibits the development of colitis. These results also indicate T-cell activation is a critical step toward the development of intestinal inflammation in autoimmune colitis.

In this study, we demonstrated TRAIL suppressed colitogenic T-cell activation, and prevented development of gut inflammation in this transfer-induced colitis model (Fig. 5). However, it is possible that a polyclonal activation of CD4 T cells prior to adoptive transfer into a lymphopenic host, may differ from those of adoptively transferred naïve CD4 T cells. We have used an adoptive transfer of TRAIL-treated naïve CD4+ CD45RB(hi) T cells without prior activation. The results showed that TRAIL-treated CD4+ CD45RB(hi) T cells exhibited later-onset and less severe development of colitis. In addition, the accumulation of CD4 T cells in the inflamed guts significantly reduced in the TRAIL-treated group (Supplementary Fig. S5), suggesting TRAIL inhibits the activation of the colitogenic T cells. In order to further address why TRAIL produced by the recipient non-T cells is not sufficient to similarly attenuate the colitogenic activity of transferred T cells, we also used TRAIL KO mice in DSS-induced colitis model. The TRAIL deficiency mice did not exacerbate colonic inflammation (Supplementary Fig. S6). These results suggest endogenous TRAIL produced by the recipient non-T cells is not sufficient to attenuate the colitogenic activity of activated T cells.

Previous studies demonstrated that TRAIL inhibited T-cell activation in an apoptosis-independent manner in autoimmune arthritis and encephalitis.^10,11^ In support of this, in the present study, TRAIL-treated T cells significantly suppressed the development of colitis and gut inflammation, indicating that TRAIL inhibited effector T-cell functions in the pathogenesis of autoimmune colitis. On the other hand, it has been well addressed that regulatory T cells (Tregs) are critical in the regulation of immune responses in many autoimmune diseases. Recent studies of IBD demonstrated that Th17 and Treg plasticity is crucial to the pathogenesis of IBD.^29–31^ However, the effects of TRAIL on the differentiation of T-cell subsets and their interactions in IBD are still unknown. In the T-cell transfer colitis model, both Th1 and Th17 effector T-cell responses contributed to colonic inflammation.^32–34^ In contrast, adoptive transfer of Tregs could protect recipient mice from colonic inflammation.^35^ Recent results from mouse models of IBD suggested that T cell plasticity, in particular, the Th17-Treg axis, plays an important role in regulating immune responses in the intestines.^31^ These findings suggest that TRAIL may also have a role in regulating proinflammatory T cells and Tregs. The results in Supplementary Fig. S7 showed that TRAIL did not induce Treg cell differentiation in the DSS-induced colitis model, suggesting TRAIL suppresses autoimmune inflammation via inhibiting T cell activation, but not by induction of regulatory T cells (Supplementary Fig. S7). Although it is still not able to exclude the possibility that TRAIL treatment might induce other regulatory cells, such as MDSCs to regulate colitis, the results from adoptive transfer-induced colitis model clearly demonstrated TRAIL suppressed T cell activation and further prevented development of gut inflammation. All these results indicate TRAIL directly inhibits colitogenic T cells in the development of colon inflammation.

In summary, we demonstrated that TRAIL significantly suppresses gut inflammation and disease severity. This implies a novel immunoregulatory role of TRAIL/TRAIL-R interactions in the pathogenesis of autoimmune colitis. Our results provide evidence that TRAIL effectively inhibits colonic T activation and suppresses autoimmune colitis, and sheds light on future therapeutic applications for targeting TRAIL/TRAIL-R in human IBD.

## Methods

### Animals

Wild-type (WT) C57BL/6 mice (female, 6 weeks old) and Rag1 KO mice (female, 6 weeks old) were housed under specific pathogen-free conditions and provided with standard food and water. TRAIL-R KO mice (C57BL/6 background, female, 6 weeks old) were obtained from Henning Walczak (UCL Cancer Institute, University College, London, UK).^36^ All animal work was conducted according to guidelines of the Association for Assessment and Accreditation of Laboratory Animal Care. All animal experiments were approved by the Animal Ethics Committee of the National Taiwan University Medical Center.

### Induction and assessment of DSS-induced colitis

To create DSS-induced colitis, mice (female, 6 weeks old) were treated with 2.5% DSS (36–50 kDa; MP Biomedicals, Santa Ana, CA) in filter-purified and sterilized drinking water for 5 days, followed by 2 days of normal water. The amount of DSS water consumed was recorded for each treatment group to ensure that all mice consumed similar amounts. Control mice (female, 6 weeks old) were given normal drinking water. Mice were monitored for weight loss and clinical manifestations (rectal bleeding and diarrhea) every day. On the seventh day, mice were sacrificed, and their colons were removed followed by weight and length measurements and a histological examination. Histological features, including cellular infiltrates, goblet cell loss, and increased epithelial proliferation, were evaluated. Hematoxylin and eosin (H&E)-stained sections were scored according to a previously described scoring system^37^ by two blinded observers. A cumulative scale with a maximum score of ten was used. Three parameters were assessed: (1) the severity of inflammation (0, none; 1, slight; 2, moderate; and 3, severe); (2) depth of injury (0, none; 1, mucosal; 2, mucosal and submucosal; and 3, transmural); and (3) crypt damage (0, none; 1, basal one-third damaged; 2, basal two-thirds damaged; 3, only surface epithelium intact; and 4, complete loss of crypts and epithelium).

### Purification of TRAIL

Recombinant TRAIL proteins were purified as described previously.^38^ In brief, the coding portion of the extracellular portion of the TRAIL molecule (amino acids 95–281) was subcloned into a pRSET(B)-His vector (Invitrogen, Groningen, the Netherlands) and expressed in an *Escherichia coli* expression system. The His-TRAIL fusion protein was purified by metal chelate column chromatography using Ni-NTA resin, according to the manufacturer’s recommendations (Qiagen, Hilden, Germany) and dialyzed. Lipopolysaccharide endotoxin was further removed from the purified TRAIL using an Acrodisc syringe filter (Pall, Port Washington, NY) and reached the targeted endotoxin level of <0.1 EU/ml as determined by a Pierce LAL Chromogenic Endotoxin Quantitation Kit (Thermo Fisher Scientific, Waltham, MA).

### Histological analysis

On day 7 in the DSS-induced colitis model or day 60 in the T-cell transfer colitis model, mice were sacrificed and their colons were removed. Colonic tissues were fixed with 4% formalin for 12 h and embedded in paraffin, and serial paraffin sections (5 µm) were stained with H&E and an anti-CD3 immunohistochemical Ab to assess tissue inflammation and T cell infiltration.

### Cytokine and chemokine arrays

To detect cytokines and chemokines of colon tissues from control mice and mice with DSS-induced colitis that underwent vehicle or TRAIL treatment, cytokine and chemokine arrays were assayed according to the manufacturer’s protocol (R&D Systems, Minneapolis, MN). In brief, mouse colon tissues lysates were added to nitrocellulose membranes spotted with selected cytokine and chemokine capture Abs, followed by mixing with a cocktail of biotinylated detection Abs. Streptavidin–horseradish peroxidase (HRP) and chemiluminescent detection reagents were sequentially added to visualize the dots on developed X-ray film. The average pixel intensity on developed film was analyzed by ImageJ vers. 1.5.

### Enzyme-linked immunosorbent assay (ELISA) for cytokines

Approximately, 200 mg of mouse distant colon tissue was homogenized and lysed using the PhosphoSafe extraction reagent (Merck Millipore, Darmstadt, Germany). Cytokine levels of colon tissue lysates, including interleukin (IL)-33 (R&D Systems), TNF-α (BioLegend, San Diego, CA), interferon (IFN)-γ (BioLegend), IL-17 (BioLegend), and IL-10 (BioLegend), were determined by ELISA kits according to the manufacturer’s instructions.

### Terminal deoxynucleotidyl transferase dUTP nick end labeling staining

To evaluate apoptotic cells within the inflamed colon, three unstained sections corresponding to the H&E-stained colon tissue slides were deparaffinized, and further stained using a TACS TdT-DAB In Situ Cell Death Detection Kit (Trevigen, Gaithersburg, MD) following the manufacturer’s instructions.

### Isolation of laminar propria cells

Mouse colon tissues were minced and gently shaken in Hanks’ balanced salt solution (HBSS) supplemented with 5 mM EDTA and 10 mM HEPES for 20 min at 37 °C. Colon fragments were then digested with collagenase VIII (0.5 mg/ml), dispase II (0.5 mg/ml), and DNase I (40 μg/ml) for 1 h at 37 °C. After filtration through a 100-μm nylon mesh, the isolated laminar propria cells were washed with HBSS and centrifuged at 300 g for 10 min. The cell pellet was collected for downstream applications.

### In vitro cell apoptosis detection

Colonic epithelial cells were isolated as previously described,^39^ and colonic CD4^+^ and CD8^+^ T cells as well as CD11c^+^ dendritic cells were further isolated from laminar propria cells using magnetic-activated cell sorting (MACS) beads (STEMCELL Technologies, Vancouver, Canada). Isolated cells were treated with various concentrations of TRAIL for 24 h at 37 °C. To detect apoptotic cells, cytoplasmic histone-associated DNA fragments from each treatment group were measured with the Cell Death Detection ELISA PLUS system according to the manufacturer’s protocol (Roche Mannheim Biochemicals, Mannheim, Germany). In brief, cells were lysed, and lysates were collected and mixed with an immunoreagent for 2 h, followed by reaction with a substrate solution in the dark until color had developed. The reaction was quantified using spectrophotometry at 405 nm.

### T-cell transfer colitis model

Splenic CD4^+^/CD25^−^ T cells from WT or TRAIL-R KO mice were isolated using MACS beads (STEMCELL Technologies). Isolated cells were treated with anti-CD3 (1 µg/ml) and anti-CD28 (1 µg/ml) Abs in the presence or absence of TRAIL (10 µg/ml) for 24 h at 37 °C. Subsequently, 5 × 10^5^ cells were transferred into Rag1 KO mice via an intraperitoneal injection. Body weight loss and clinical symptoms were recorded daily.

### Statistical analysis

All data are expressed as the mean ± standard deviation. Statistical significance was determined by Student’s *t* test for unpaired samples. For the analysis of KO mice data, the Mann–Whitney *U* test was performed to assess differences between the WT and KO mice groups. A *p* value of <0.05 was defined as statistically significant. All analyses were conducted using SPSS software, version 16.0 (SPSS, Chicago, IL).

## Supplementary information

Supplementary Information
