# Supplementary material for: TRAIL suppresses gut inflammation and inhibits colitogeic T-cell activation in experimental colitis via an apoptosis-independent pathway
Source: Mucosal Immunol. 2019 May 11;12(4):980–9. doi: 10.1038/s41385-019-0168-y (PMC7746525; doi:10.1038/s41385-019-0168-y)
Supplement: Supplementary file 1 — Supplementary Information [file 41385_2019_168_MOESM1_ESM.pdf]

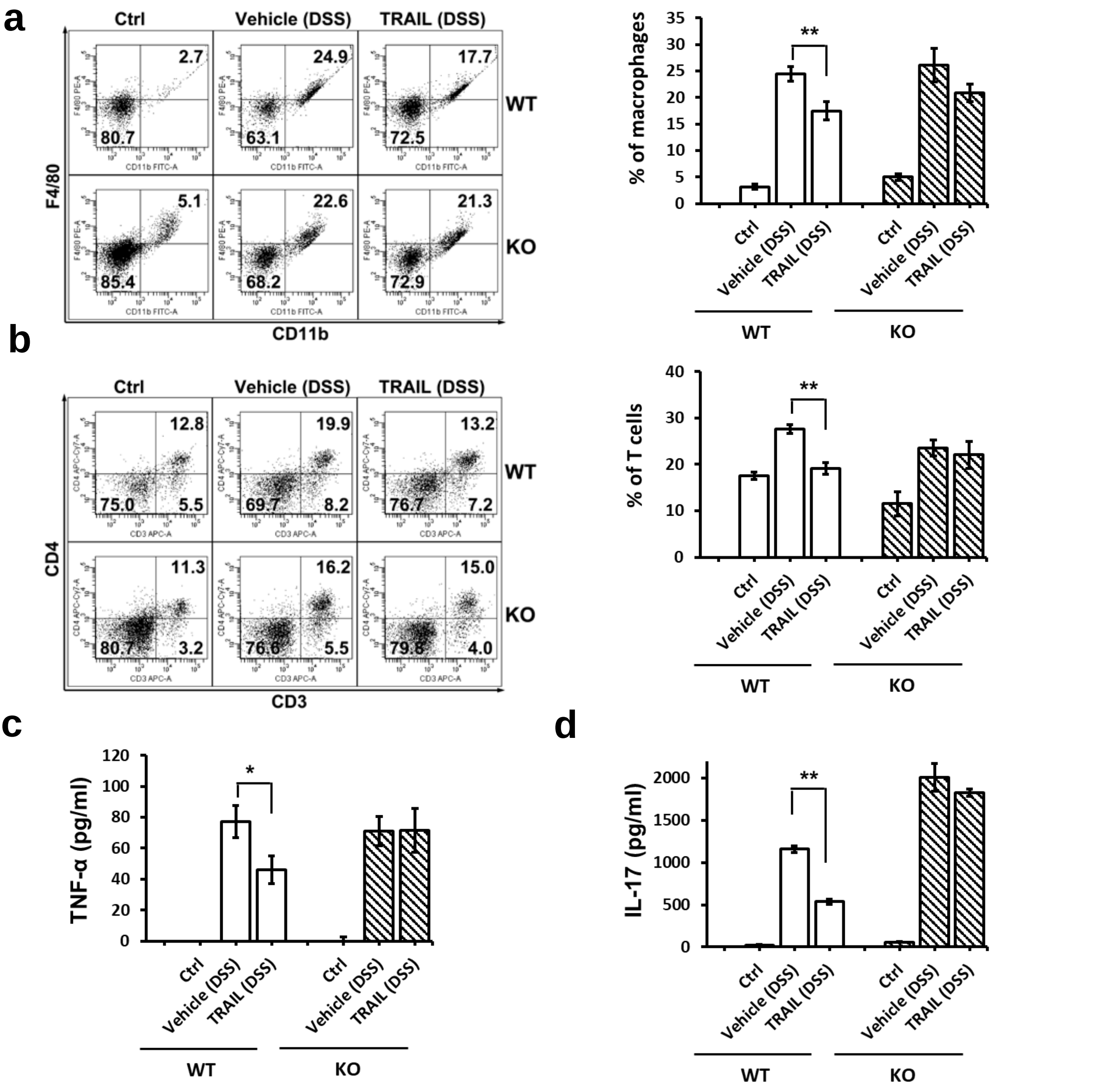

Figure S1. TRAIL inhibits the recruitment of both innate and adaptive immune cells into inflamed guts in mice with DSS-induced colitis.

Seven days after the DSS induction with or without TRAIL treatment, the frequency of (a) macrophage and (b) CD4 T cells in isolated colonic lamina propria (LP) cells from mice in the indicated groups were determined by flow cytometry. Representative plots (left panel) and quantitative data (right panel) are shown. (c) The macrophages and (d) CD4 T cells were isolated from LP cells in the indicated group and cultured in 96-well flat-bottomed microtiter plates (2×10^5 cells/well) for 24 h. The supernatants were collected and assayed for TNF-α and IL-17 production by ELISA , respectively. The data are shown as the mean ± SD of quadriplicate samples. ** $p < 0.01$ by non-parametric Mann–Whitney U-test.

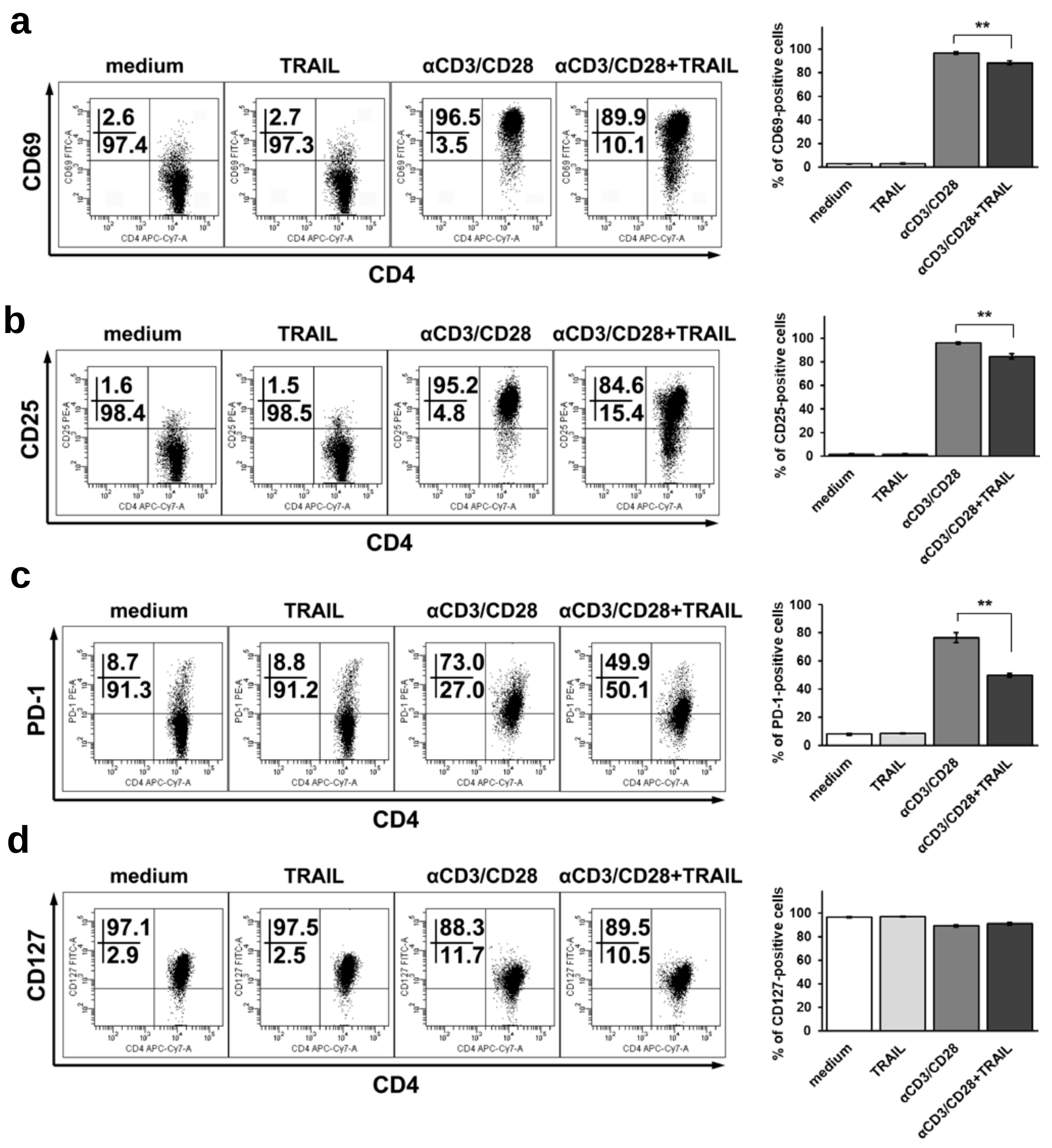

Figure S2. TRAIL inhibited expression of T cell activation markers.
2×10^6 primary splenic CD4 T cells from C57BL/6 mice were stimulated with medium, an anti-CD3 antibody (Ab; 1 µg/ml) plus anti-CD28 Ab (1 µg/ml), and TRAIL (10 µg/ml), or a combination of anti-CD3/anti-CD28 Abs and TRAIL. The cells were stained with (a) anti-CD69 Ab, (b) anti-CD25 Ab, (c) anti-PD-1 Ab or (d) anti-CD127 Ab and analyzed by flow cytometry. Representative figures for each group are shown (left panel) and quantified (right panel) from three independent experiments in each group. ** $p < 0.01$ by non-parametric Mann–Whitney U-test.

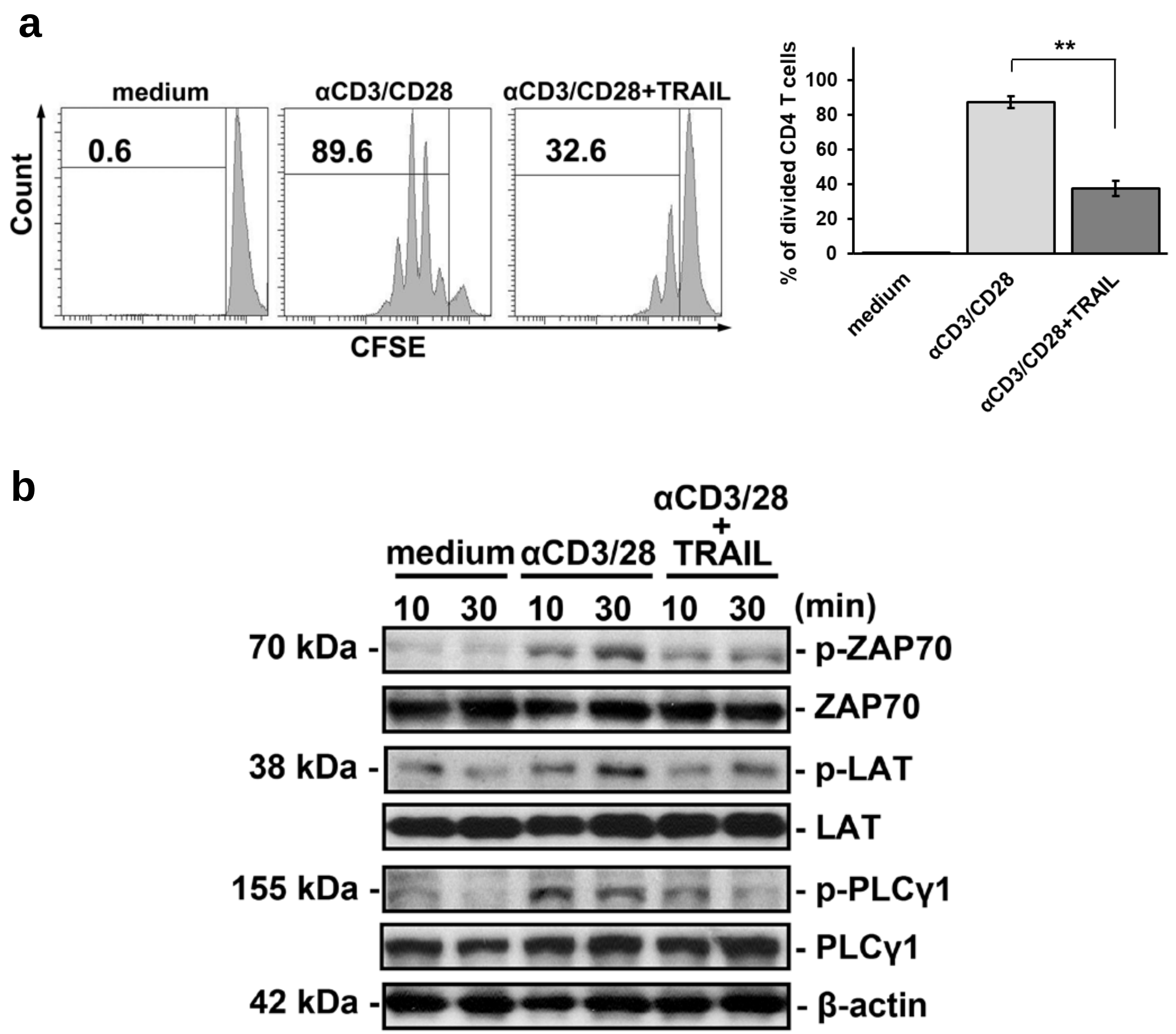

Figure S3. TRAIL suppressed T cell activation and inhibited T cell receptor signaling. (a) 2×10^6 primary splenic CD4 T cells from C57BL/6 mice were labeled with CFSE and cultured in 96-well flat-bottomed microtiter plates coated with medium or anti-CD3 Ab (1 μg/ml) and anti-CD28 Ab (1 μg/ml) in the presence or absence of TRAIL (10 μg/ml) for 5 days. Representative figures for each group are shown (left panel) and quantified (right panel) from three independent experiments in each group. ** $p < 0.01$ by non-parametric Mann–Whitney U-test (b) 2×10^6 primary splenic CD4 T cells from C57BL/6 mice were stimulated at the indicated time point with medium, an anti-CD3 and anti-CD28 Abs (1 μg/ml) in the presence or absence of TRAIL (10 μg/ml). Lysates of each group were prepared and immunoblotted with anti-phospho-ZAP70 (Tyr319), anti-ZAP70, anti-phospho-LAT (Tyr191), anti-LAT, anti-phospho-PLCγ1 (Tyr783), anti-PLCγ1, and anti-actin Abs. Blots are representative of three independent experiments performed.

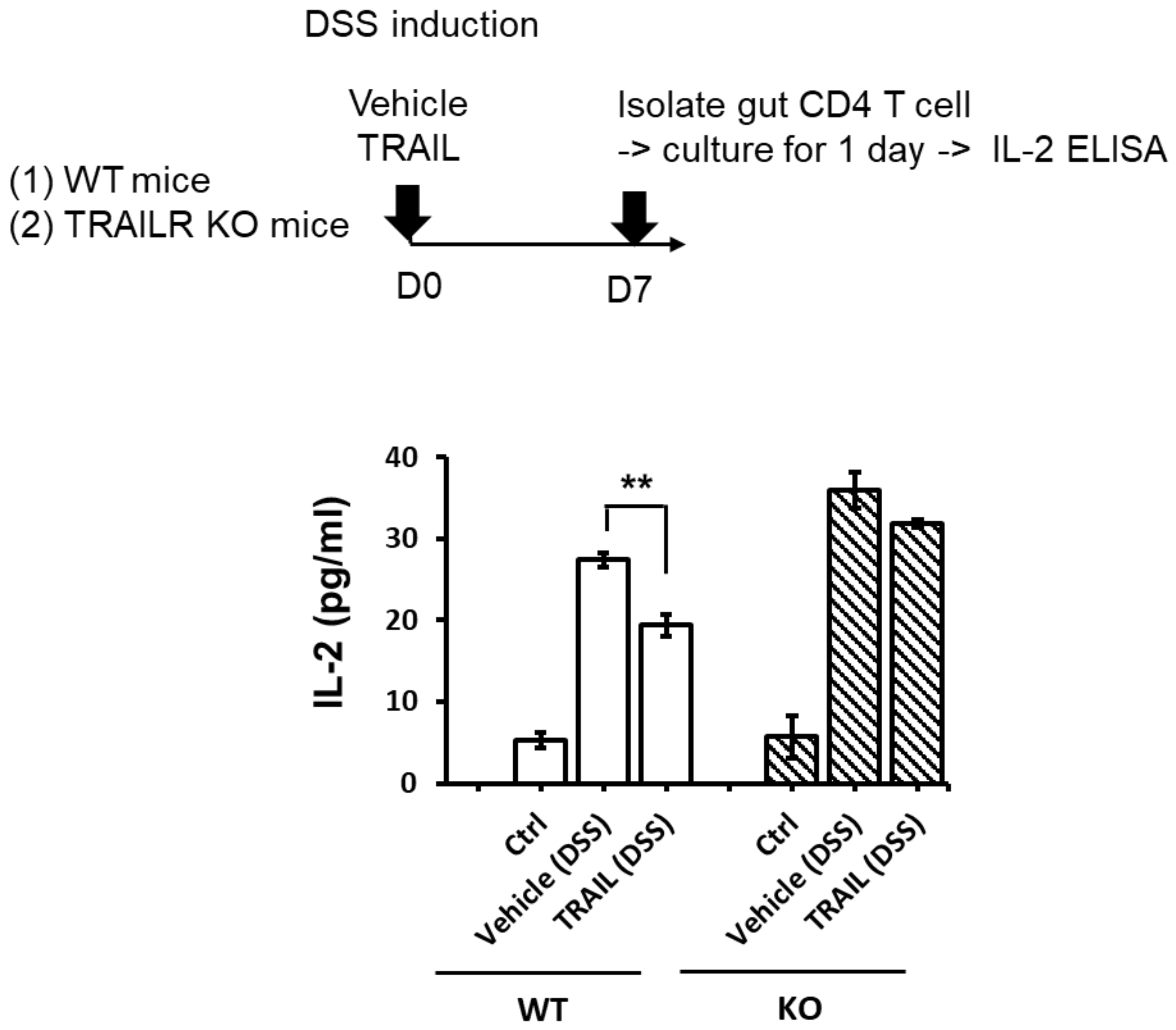

Figure S4. TRAIL suppressed IL-2 production in T cells from inflamed guts.
Wild-type C57BL/6 (WT) mice and TRAIL-R knockout (KO) mice were fed 2.5% DSS in sterilized drinking water for 5 days (days 0~5), followed by 2 days of normal water (days 6 and 7) and treated with either the vehicle (200 µl/mouse/day, i.p.) or TRAIL (50 µg/mouse/day, i.p.) from day 0. Control mice were given normal drinking water only. On day 7, CD4 T cells were isolated from the inflamed guts and cultured in 96-well flat-bottomed microtiter plates (2×10^5 cells/well) for 24 h. The supernatants were collected and assayed for IL-2 production by ELISA. The data are shown as the mean ± SD of quadriplicate samples. ** $p < 0.01$ by non-parametric Mann–Whitney U-test.

a

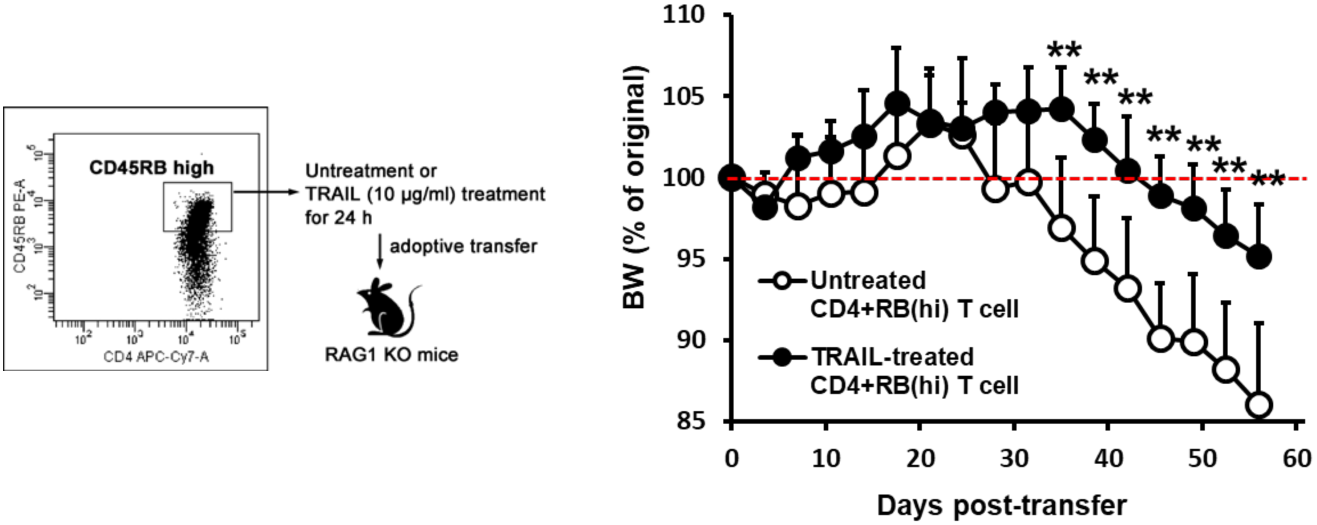

b

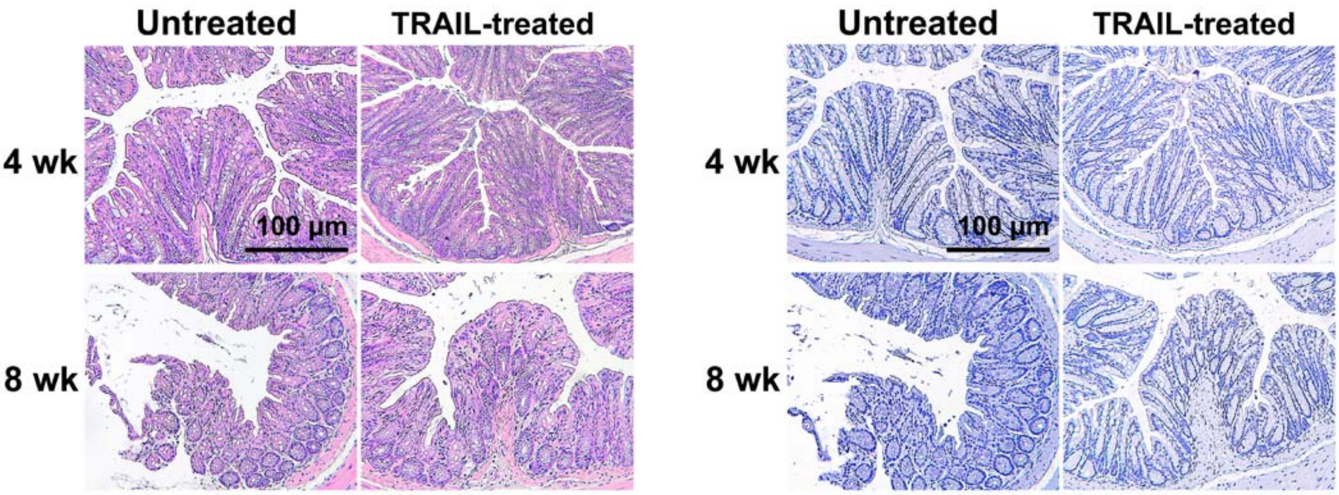

Figure S5. TRAIL reduced colonic inflammation and accumulation of T cells in the inflamed guts after adoptive transfer.

Splenic $CD4^+CD45RB^{high}$ T cells from C57BL/6 mice were sorted and treated with or without TRAIL (10 μg/ml) for 24 h, following adoptive transfer into Rag1 knockout recipients (5×10^5 cells/recipient, i.p.). (a) Body weight changes (% original body weight) in each group were recorded and are presented as the mean ± SD (n=10 in each group). ** $p < 0.01$, Mann-Whitney U-test, compared to WT mice treated with the vehicle. (b) Representative H&E stain (left panel) and TUNEL stain (right panel) in each group on 4 week and 8 week are shown.

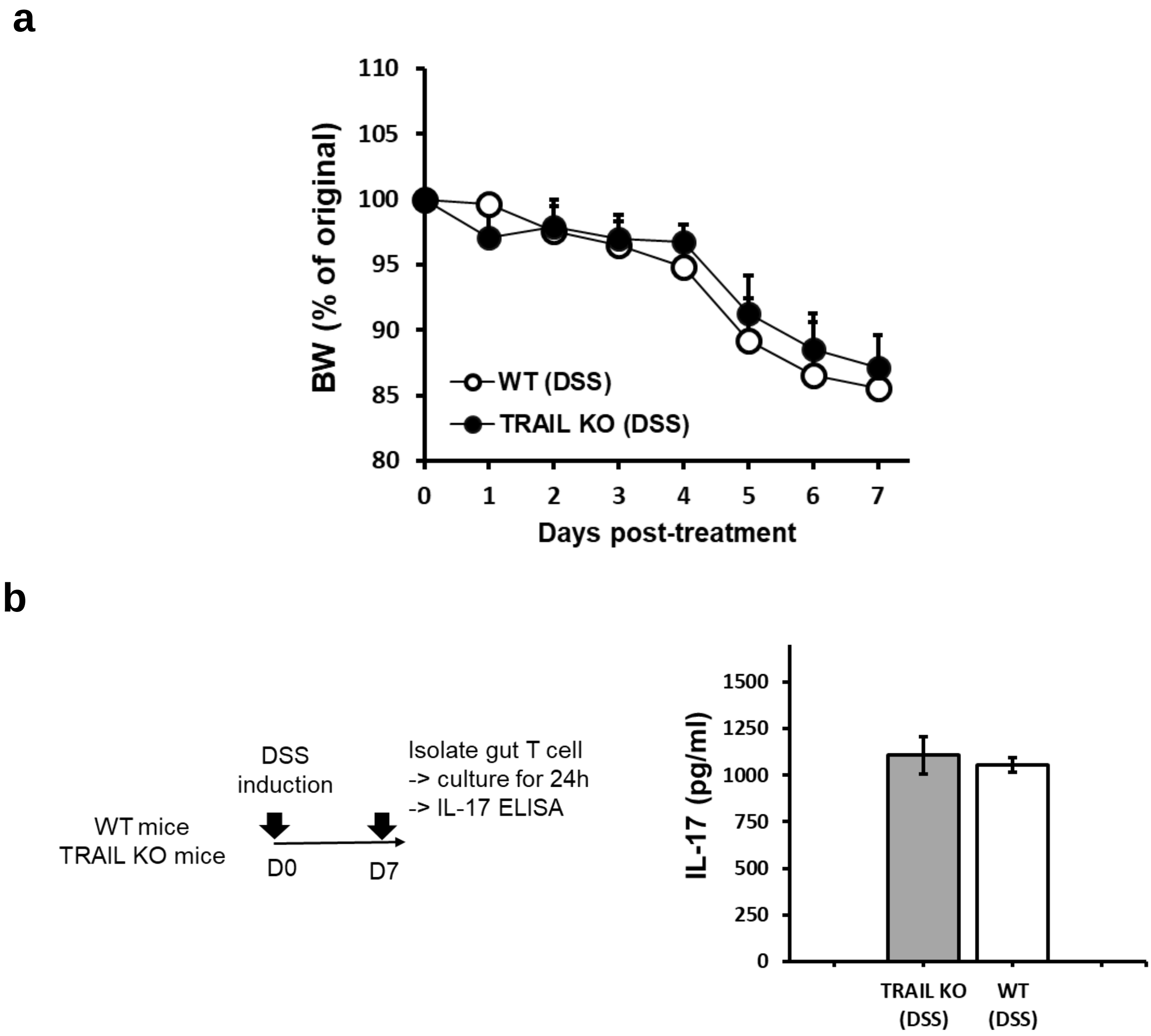

Figure S6. TRAIL deficiency did not exacerbate colonic inflammation.
Wild-type C57BL/6 (WT) mice and TRAIL knockout (TRAIL KO) mice were fed 2.5% DSS in sterilized drinking water for 5 days (days 0~5), followed by 2 days of normal water (days 6 and 7). (a) Body weight changes (% original body weight) in each group were recorded and are presented as the mean ± SD (n=10 in each group). (b) On day 7, , CD4 T cells were isolated from the inflamed guts and cultured in 96-well flat-bottomed microtiter plates (2×10^5 cells/well) for 24 h. The supernatants were collected and assayed for IL-17 production by ELISA. The data are shown as the mean ± SD of quadriplicate samples.

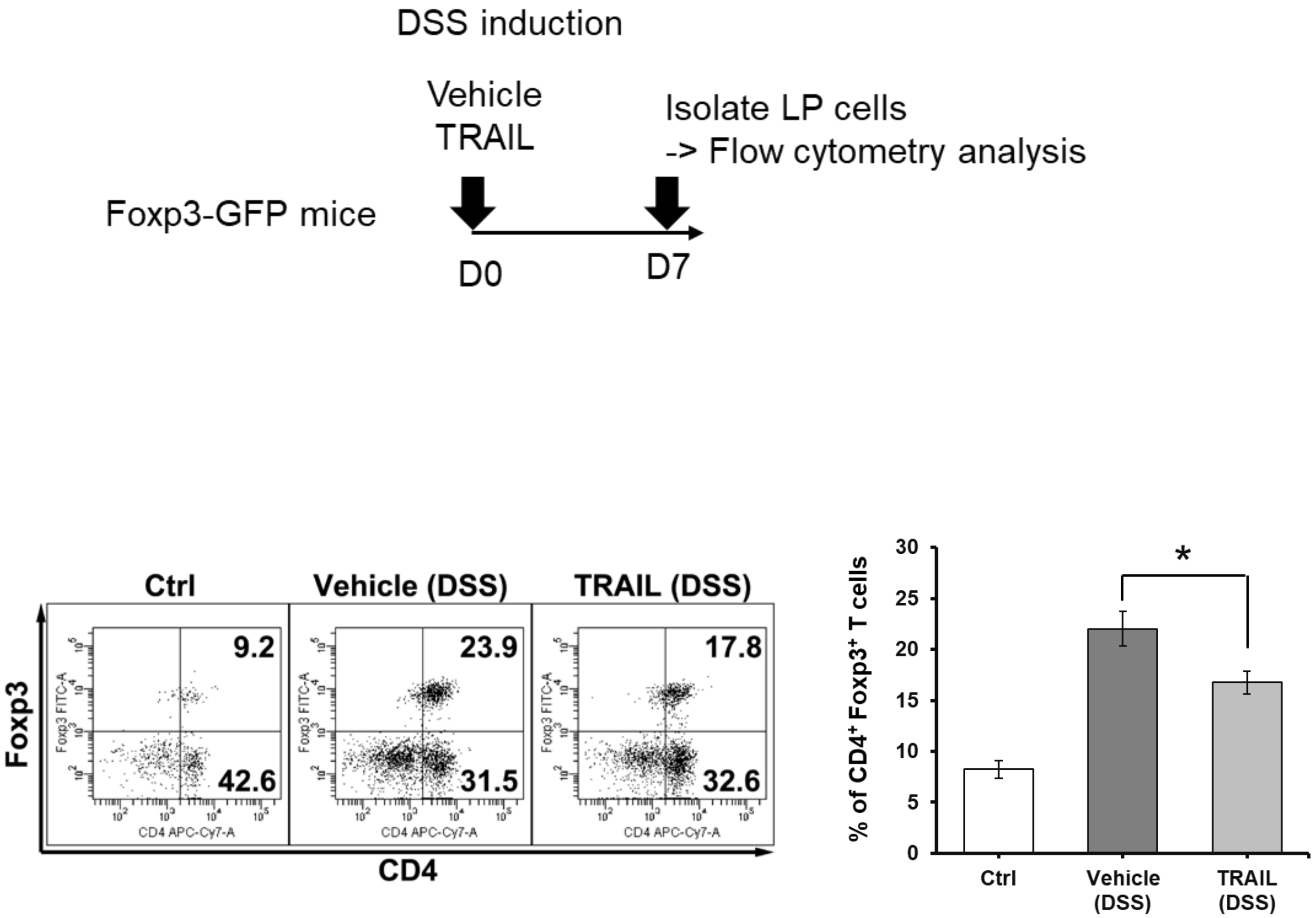

Figure S7. TRAIL did not induce regulatory T cells in DSS-induced colitis.
Foxp3^{GFP} mice were fed 2.5% DSS in sterilized drinking water for 5 days (days 0~5), followed by 2 days of normal water (days 6 and 7) and treated with either the vehicle (200 µl/mouse/day, i.p.) or TRAIL (50 µg/mouse/day, i.p.) from day 0. Control mice were given normal drinking water only. On day 7, lamina propria (LP) cells were isolated from the inflamed guts, stained with anti-CD3 and anti-CD4 Ab, and analyzed by flow cytometry. Representative plots (left panel) and quantitative data (right panel) are shown. * $p < 0.01$ by non-parametric Mann–Whitney U-test.
